# The transition from field emission to collisional space-charge limited current with nonzero initial velocity

**DOI:** 10.1038/s41598-023-41615-2

**Published:** 2023-09-04

**Authors:** Lorin I. Breen, Amanda M. Loveless, Adam M. Darr, Keith L. Cartwright, Allen L. Garner

**Affiliations:** 1 School of Health Sciences, Purdue University, West Lafayette, IN 47907 USA; 2 Department of Agricultural and Biological Engineering, Purdue University, West Lafayette, IN 47907 USA; 3 School of Nuclear Engineering, Purdue University, West Lafayette, IN 47907 USA; 4 Present Address: Sandia National Laboratories, Albuquerque, NM 87123 USA; 5 Elmore Family School of Electrical and Computer Engineering, West Lafayette, IN 47907 USA

**Keywords:** Plasma physics, Scaling laws, Electrical and electronic engineering

## Abstract

Multiple electron emission mechanisms often contribute in electron devices, motivating theoretical studies characterizing the transitions between them. Previous studies unified thermionic and field emission, defined by the Richardson-Laue-Dushman (RLD) and Fowler–Nordheim (FN) equations, respectively, with the Child-Langmuir (CL) law for vacuum space-charge limited current (SCLC); another study unified FN and CL with the Mott-Gurney (MG) law for collisional SCLC. However, thermionic emission, which introduces a nonzero injection velocity, may also occur in gas, motivating this analysis to unify RLD, FN, CL, and MG. We exactly calculate the current density as a function of applied voltage over a range of injection velocity (i.e., temperature), mobility, and gap distance. This exact solution approaches RLD, FN, and generalized CL (GCL) and MG (GMG) for nonzero injection velocity under appropriate limits. For nonzero initial velocity, GMG approaches zero for sufficiently small applied voltage and mobility, making these gaps always space-charge limited by either GMG at low voltage or GCL at high voltage. The third-order nexus between FN, GMG, and GCL changes negligibly from the zero initial velocity calculation over ten orders of magnitude of applied voltage. These results provide a closed form solution for GMG and guidance on thermionic emission in a collisional gap.

The continuing diversification in size, pressure, temperature, and phases of matter in electronic devices motivates the unification of various electron emission models to improve device design and operation^1–4^. Characterizing electron emission and the transitions between mechanisms is critical for numerous applications, including directed energy, high power vacuum electronics, time-resolved microscopy, and x-ray systems^3^. Moreover, electron emission plays a critical role in discharge generation for microscale and smaller gaps, where field and/or thermionic emission may strip sufficient electrons from the cathode due to high electric fields and/or temperature, respectively, to induce discharge^3,5–7^.

However, practical devices may not operate in a single electron emission regime, motivating the characterization of the transition between these mechanisms^3^. Increasing the emission current of a device, regardless of the emission mechanism, ultimately causes electron emission to become limited due to the presence of too much charge in the gap^8–10^. Lau et al. solved for the transit time of a single electron emitted from the cathode by field emission to derive an exact solution for the current density that accounted for space-charge^8^. In the limits of high applied voltage $V$ and/or small gap distance $D$, this solution reduced to the space-charge limited current (SCLC) in vacuum, the Child-Langmuir (CL) law^11,12^, given by

1 $${J}_{CL}=\frac{4\sqrt{2}}{9}{\varepsilon }_{0}\sqrt{\frac{e}{m}} \frac{{V}^{3/2}}{{D}^{2}},$$

where ${\varepsilon }_{0}$ is the permittivity of free space, $e$ is electron charge, and $m$ is electron mass. In the limits of low $V$ or large $D$, the solution approached the Fowler–Nordheim (FN) equation for field emission, given by^13–15^

2 $${J}_{FN}=\frac{{A}_{FN}{E}_{s}^{2}}{{t}^{2}\left(y\right)}\mathrm{exp}\left[-{B}_{FN}v\left(y\right)/{E}_{s}\right],$$

where

3 $$v\left(y\right)\approx 1-\frac{{y}^{2}}{3}\left(3-\mathrm{ln}y\right),$$

4 $$t\left(y\right)\approx 1+\frac{{y}^{2}}{9}\left(1-\mathrm{ln}y\right),$$

${E}_{s}$ is the electric field at the cathode, $y={\Phi }^{-1}\sqrt{4Qe{E}_{s}}$ where $Q={e}^{2}/\left(16\pi {\epsilon }_{0}\right)$, ${A}_{FN}={e}^{3}/\left(16{\pi }^{2}\hslash\Phi \right)$ and ${B}_{FN}=4\sqrt{2m{\Phi }^{3}}/\left(3\hslash e\right)$ are FN constants, $\Phi$ is the electrode work function, and $\hslash$ is the reduced Planck’s constant. Table 1 summarizes the values of key physical parameters used in these equations and throughout this paper.

**Table 1:** Typical values of physical parameters.

Parameter	Quantity	Value
${A}_{FN}$	Fowler Nordheim coefficient (at 4.5 eV)	$3.44\times {10}^{-7} \mathrm{A }{\mathrm{V}}^{-2}$
${B}_{FN}$	Fowler Nordheim coefficient (at 4.5 eV)	$6.55\times {10}^{10} \mathrm{V }{\mathrm{m}}^{-1}$
$e$	Electron charge	$1.602\times {10}^{-19}\mathrm{ C}$
$m$	Electron mass	$9.11\times {10}^{-31}\mathrm{ kg}$
${k}_{B}$	Boltzmann’s constant	$1.38\times {10}^{-23} \mathrm{J }{\mathrm{K}}^{-1}$
${\varepsilon }_{0}$	Permittivity of vacuum	$8.854\times {10}^{-12} \mathrm{F }{\mathrm{m}}^{-1}$
$\hslash$	Reduced Planck’s constant	$1.05\times {10}^{-34}\mathrm{ J s}$
$Q$	Fowler Nordheim constant	$5.77\times {10}^{-29} \mathrm{J m}$
$\Phi$	Work function	$4.5 \mathrm{eV}$
${E}_{0}$	Electric field scaling constant	$6.55\times {10}^{10} \mathrm{V }{\mathrm{m}}^{-1}$
${F}_{0}$	Force scaling constant	$1.05\times {10}^{-8} \mathrm{N}$
${T}_{0}$	Temperature scaling constant	$5.22\times {10}^{4} \mathrm{K}$
${t}_{0}$	Time scaling constant	$3.93\times {10}^{-16} \mathrm{s}$
${\phi }_{0}$	Voltage scaling constant	$116.5 \mathrm{V}$
${v}_{0}$	Velocity scaling constant	$4.53\times {10}^{6} \mathrm{m }{\mathrm{s}}^{-1}$

Recasting these equations in nondimensional variables yielded a universal (true for any diode geometry) set of equations and matching the resulting asymptotic solutions indicated the transition from a field emitting diode to a space-charge limited one^8^. Note that this matching invalidates the assumptions inherent in the fundamental equations since CL is commonly derived for ${E}_{s}=0$, while FN requires nonzero ${E}_{s}=V/D$. Thus, characterizing electron emission near this “nexus” necessitates solving the full solution that accounts for all relevant physics.

Similar asymptotic approaches have been applied to characterize other emission and circuit phenomena. By introducing electron mobility $\mu$ into the electron force law to account for collisions, Benilov assessed the transition from CL to SCLC with collisions^16^, or the Mott-Gurney (MG) law, given by^17^

5 $${J}_{MG}=\frac{9}{8}\mu {\varepsilon }_{0}\frac{{V}^{2}}{{D}^{3}},$$

where $\mu$ is the electron mobility in the gas. Additional applications of this approach to assess field emission, SCLC, and resistive dissipation include the transitions between CL, FN, and Ohm’s law^18^; CL, FN, and MG^19^; and CL, FN, MG, and Ohm’s law^20^. This approach has also been applied to include the enhancement in quantum SCLC at small gaps with the transition to field emission driven microdischarges at large gaps^21^ and the transitions between FN and SCLC in liquids and during the transition in phase from liquid to gas^22^. This approach of developing full analytic theories coupling relevant electron emission sources and assessing the transition between dominant mechanisms, referred to more broadly as “nexus theory”^4^, may be extended to other electron emission source mechanisms. For instance, Darr et al.^23^ replaced FN with the general thermal-field (GTF) emission equation^2,24–26^, which couples field and thermionic emission, to derive exact and asymptotic solutions linking FN, CL, and the Richardson-Laue-Dushman (RLD) solution for thermionic emission, given by^27^

6 $${J}_{RLD}={A}_{RLD}{T}^{2}\mathrm{exp}\left(\frac{\Phi }{{k}_{B}T}\right),$$

where ${{T}}$ is the cathode temperature, ${k}_{B}$ is the Boltzmann constant, and ${A}_{RLD}=em/\left(2{\pi }^{2}{\hslash }^{3}\right)$. Darr et al. extended previous studies examining the transition from RLD to CL using Miram curves^28–33^ by incorporating FN and showing that an increasing contribution of field emission could soften the characteristic “knee” during the transition to CL. Lang et al.^34^ replaced the GTF equation with the general thermo-field photoemission (GTFP) equation^24,25^ to include the transition to the Fowler-DuBridge (FD) equation for photoemission^2,35^. In addition to providing a detailed step-by-step process for developing nexus phase space plots showing the device conditions necessary for the transitions between FD, RLD, FN, MG, CL, and Ohm’s law, Lang et al. derived exact solutions for the current density as a function of voltage and demonstrated the transitions to FD, RLD, FN, and CL under appropriate asymptotic conditions of temperature, gap distance, voltage, and laser frequency^34^.

Nexus theory may also be applied to assess variations in electron emission regime for rough electrodes. A recent study applied nexus theory to a fractional dimensional model to derive the conditions for transitions between FN, MG, and CL for different conditions of surface roughness^36^. Such a model may be extended to the condition here combining RLD with FN, MG, and CL by following the steps outlined in that study; however, it would also be necessary to extend the GTF representation of combined electron emission to fractional dimensions to derive the solution for the exact current density in the diode.

Although a given emitter may realistically exhibit multiple mechanisms due to field and temperature non-uniformities, the present study considers a one-dimensional (1D) system assuming all electron emission behavior along the cathode follows a common mechanism (or combination of mechanisms). Using 1D nexus theory simplifies the assessment of emitter behavior, elucidating inter-mechanism transitions based on operating system conditions.

While several studies have applied nexus theory to assess numerous transitions, none have explicitly considered the transition between RLD and MG. While this transition has been examined using nexus theory by matching the RLD and MG^23^, an exact solution fully coupling collisions and the GTF or GTFP source has not yet been conducted. While seemingly of academic interest, this assessment has practical implications in device physics. Although many vacuum electronics devices use cold cathodes (field emission with the electrons emitted with zero velocity)^35,37,38^, many use thermionic cathodes (electrons emitted with nonzero velocity)^38–40^. This motivated the earlier study linking RLD with FN and CL^23^. Less often considered theoretically, although motivating the previous study linking MG with FN and CL^19^, is that vacuum devices often do not operate in perfect vacuum. Pressures of 10^–5^ Torr can dramatically degrade field emission^41^ and the failure rate of emitters increases with increasing pressure^42^. Alternatively, at atmospheric pressure for microscale gaps, strong electric fields strip electrons off the cathode by field emission to reduce breakdown with decreasing gap size^5^. Reducing gap size to nanoscale causes space-charge to begin to limit the current prior to breakdown^7^. Since thermionic emission may also play a role in micro- or nano- scale breakdown^6^, this physics is also relevant for higher pressures; one example being high altitude discharge. For instance, when attempting to define “high pressure” and “low pressure” cutoffs for choosing the correct model to describe a device, it can be helpful to know the “midpoint pressure” as defined by nexus theory involving MG. This could be especially useful in thermionic diodes, to define a threshold for temperature-induced outgassing that may change device operation^43–47^. Hanquist et al. addressed high heating rates on hypersonic vehicles and developed a model combining SCLC with thermionically emitted electrons at the surface to include transpiration cooling^48^.

The other challenge is that when examining the nexuses of SCLC with RLD, one must also consider the implications of nonzero injection velocity on SCLC. The most widely-cited derivation of 1D, planar SCLC with nonzero electron injection velocity was derived by Jaffé^49–51^ as

7 $$\frac{{J}_{Jaff \acute{e}}}{{J}_{CL}}= {\left[{\left(\frac{m{v}_{0}^{2}}{2eV}\right)}^{1/2}+{\left(1+\frac{m{v}_{0}^{2}}{2eV}\right)}^{1/2}\right]}^{3},$$

where ${v}_{0}$ is electron injection velocity. Liu and Dougal derived an alternate solution, referred to as the bifurcation current density ${J}_{B}$, given by^52^

8 $$\frac{{J}_{B}}{{J}_{CL}}= {\left[{\left(\frac{m{v}_{0}^{2}}{2eV}\right)}^{3/4}+{\left(1+\frac{m{v}_{0}^{2}}{2eV}\right)}^{3/4}\right]}^{2}.$$

In (8), the electrons have zero velocity at the virtual cathode. In (7), the electric potential is shallower since the electrons maintain a nonzero velocity at the virtual cathode. From the asymptotic analysis of the transition from RLD to CL, Darr et al. derived a generalized CL law (GCL) ${J}_{GCL}$ as^23^

9 $${J}_{GCL}\approx {J}_{CL}\left(\sqrt{1+\frac{{mv}_{0}^{2}}{2eV}}-\sqrt{\frac{{mv}_{0}^{2}}{2eV}}\right){\left(\sqrt{1+\frac{{mv}_{0}^{2}}{2eV}}+2\sqrt{\frac{{mv}_{0}^{2}}{2eV}}\right)}^{2}\approx {J}_{CL}\left(1+3\sqrt{\frac{{mv}_{0}^{2}}{2eV}}\right),$$

which behaves identically to (7) in the limit of $m{v}_{0}^{2}{\left(2eV\right)}^{-1}\ll 1$. However, no readily available solution for an analogous general MG (GMG) solution for nonzero injection velocity exists to serve as an appropriate nexus equation for a thermionic cathode in a non-vacuum environment.

Thus, this paper will develop a first-principles based linkage of FN, RLD, GMG, and GCL to assess this behavior considering a 1D system. We first derive the theory starting from the electron force law coupled with the GTF and obtain the relevant asymptotic solution for GMG. We next apply the resulting asymptotic theories and exact solutions to assess nexuses under various conditions before making concluding remarks.

## Results

### Derivation

We consider a 1D, planar diode containing neutral gas with electron mobility $\mu$, the cathode at $x=0$ held at electric potential $\phi =0$, and the anode at $x=D$ held at $\phi =V$ with respect to the cathode. We assume the electron is emitted from the cathode at $x\left(0\right)=0$ with initial velocity $v\left(0\right)={v}_{0}$ and initial acceleration $a(0)=e{E}_{s}/m$. Combining Poisson’s equation with continuity, given by $J=e{n}_{e}v$, yields

10 $$\frac{{d}^{2}\phi }{d{x}^{2}}=\frac{J}{{\epsilon }_{0}v},$$

where ${n}_{e},$ $v,$ and $J$ are electron number density, electron velocity, and current density, respectively. Assuming that the current is emitted due to a combination of thermionic and field emission, we define $J$ using the GTF relation, ${J}_{GTF}={A}_{RLD}{T}^{2}N(n,s)$, where ${A}_{RLD}=(em{k}_{B}^{2})/(2{\pi }^{2}{\hslash }^{3})$, $\hslash$ is the reduced Planck constant, $n={\beta }_{T}/{\beta }_{F}$, ${\beta }_{T}=1/({k}_{B}T)$, and ${\beta }_{F}$, $s$, and $N(n,s)$ are functions of $F$ and $T$ (see Supplementary Information)^23^. The force on an electron is given by

11 $$m\frac{dv}{dt}=e\frac{d\phi }{dx}-\frac{ev}{\mu }.$$

The first term on the right-hand-side of (11) represents the force on the electron due to the electric field, while the second represents a friction term introduced by collisions. To reduce parameters and facilitate analysis, we nondimensionalize (10) and (11) by defining

12 $$\phi ={\phi }_{0}\overline{\phi }; J={J}_{0}\overline{J }; x={x}_{0}\overline{x }; t={t}_{0}\overline{t }; \mu ={\mu }_{0}\overline{\mu }; E={E}_{0}\overline{E }; v={v}_{0}\overline{v }; T={T}_{0}\overline{T }; \phi =\frac{e{\epsilon }_{0}^{2}}{m{A}_{FN}^{2}}; {J}_{0}={A}_{FN}{B}_{FN}^{2}; {x}_{0}=\frac{e{\epsilon }_{0}^{2}}{m{A}_{FN}^{2}{B}_{FN}}; {t}_{0}=\frac{{\epsilon }_{0}}{{A}_{FN}{B}_{FN}}; {\mu }_{0}=\frac{e{\epsilon }_{0}}{m{A}_{FN}{B}_{FN}}; {T}_{0}=\frac{\Phi }{{k}_{B}}; {E}_{0}={B}_{FN}; {v}_{0}=\frac{{x}_{0}}{{t}_{0}},$$

where the bars represent dimensionless parameters, terms with subscript 0 are scaling terms, and the FN coefficients are given by ${A}_{FN}={e}^{3}/(16{\pi }^{2}\hbar\Phi )$ and ${B}_{FN}=\left(4\sqrt{2m{\Phi }^{3}}\right)/(3\hbar e)$. Substituting (12) into (10) and (11) yields

13 $$\frac{{d}^{2}\overline{\phi }}{d{\overline{x} }^{2}}=\frac{\overline{J} }{\overline{v} }$$

and

14 $$\frac{d\overline{v} }{d\overline{t} }=\frac{d\overline{\phi }}{d\overline{x} }-\frac{\overline{v}}{\overline{\mu } },$$

respectively. Equations (13) and (14) are universal since all material dependence has been removed through nondimensionalization.

Differentiating (14) with respect to $\overline{x }$, considering $\overline{v }=d\overline{x }/d\overline{t }$ to change variables, and combining with (13) gives

15 $$\overline{J }=\frac{{d}^{2}\overline{v} }{d{\overline{t} }^{2}}+\frac{1}{\overline{\mu }}\frac{d\overline{v} }{d\overline{t} }.$$

Solving (15) for velocity using the initial conditions defined previously yields

16 $$\overline{v }\left(\overline{t }\right)=\overline{\mu }\left[\left(\overline{\mu }\overline{J }-\overline{E }\right)\left({e}^{-\overline{t }/\overline{\mu }}-1\right)+\overline{J }\overline{t }\right]+{\overline{v} }_{0},$$

where ${v}_{0} =\sqrt{\left({k}_{B}T\right)/{m}_{e}}$^23^, which may be written non-dimensionally as ${\overline{v} }_{0}= \sqrt{\overline{T }/26.1145}$. Integrating (16) gives electron position as

17 $$\overline{x }\left(\overline{t }\right)=\overline{\mu }\left[\left(\overline{\mu }\overline{J }-\overline{E }\right)\left(-\overline{\mu }{e}^{-\overline{t }/\overline{\mu }}-\overline{t }+\overline{\mu }\right)+\frac{\overline{J }{\overline{t} }^{2}}{2}\right]+{\overline{v} }_{0}\overline{t }.$$

At low mobility (high pressure), $\mathrm{exp}(-\overline{t }/\overline{\mu })\approx 0$, simplifying (16) and (17) to $\overline{v }(\overline{t}) \approx \overline{\mu }\left(\overline{E }+\overline{J }\overline{t }\right)+{\overline{v} }_{0}$ and $\overline{x }(\overline{t}) \approx \overline{\mu }\left(\overline{E }\overline{t }+\overline{J }{\overline{t} }^{2}/2\right)+{\overline{v} }_{0}\overline{t }$, respectively. The critical current density can then be obtained by considering the condition $\overline{x }\left(\overline{\tau }\right)=\overline{D }$. This gives the transit time as $\overline{\tau }=\chi \overline{E }/\overline{J }$, with $\chi$ simplifying to

18 $$\chi =-\frac{{\overline{v} }_{0}}{\overline{E}\overline{\mu } }+{\left(\frac{2\overline{D }\overline{J} }{{\overline{E} }^{2}\overline{\mu }}+\frac{{\overline{v} }_{0}^{2}}{{\overline{E} }^{2}{\overline{\mu }}^{2}}\right)}^{1/2}=\frac{{\overline{v} }_{0}}{\overline{E}\overline{\mu } }\left[-1+{\left(1+\frac{2\overline{D }\overline{J}\overline{\mu } }{{\overline{v} }_{0}^{2}}\right)}^{1/2}\right],$$

for $$\overline{\mu }\ll 1$$.

Instead of defining $\overline{V }$ using an energy balance equation^8^, we integrate (14) with respect to $\overline{x }$ and change variables to $\overline{t }$ to obtain^19^

19 $$\overline{V }={\left.\frac{\overline{v }{\left(\overline{t }\right)}^{2}}{2}\right|}_{0}^{\overline{\tau }}+{\int }_{0}^{\overline{\tau }}d\overline{t }\frac{\overline{v }{\left(\overline{t }\right)}^{2}}{\overline{\mu }},$$

where $\overline{v }(\overline{t })$ is defined by (16). Because $\overline{\mu }\ll 1$, the second term on the right-hand side of (19) dominates. Substituting the simplified velocity function, $\overline{v }(\overline{t})\approx\overline{\mu }\left(\overline{E }+\overline{J }\overline{t }\right)+{\overline{v} }_{0}$, and $\overline{\tau }$ into (19) gives

20 $$\overline{V }=\frac{\chi \overline{E }\left(\left(3+3\chi +{\chi }^{2}\right){\overline{E} }^{2}{\overline{\mu }}^{2}+3\left(2+\chi \right)\overline{E}\overline{\mu }{\overline{v} }_{0}+3{\overline{v} }_{0}^{2}\right)}{3\overline{J}\overline{\mu } }.$$

Neglecting higher order terms of $\overline{\mu }$, considering $\overline{V }\gg 1,$ and incorporating the definition of $\chi$ from (18) simplifies (20) to give the general MG (GMG) equation for nonzero initial velocity as

21 $${\overline{J} }_{GMG}=\frac{9\overline{\mu }{\overline{V} }^{2}}{8{\overline{D} }^{3}}\left(\frac{1}{2}-\frac{2{\overline{D} }^{2}{\overline{v} }_{0}^{2}}{3{\overline{\mu }}^{2}{\overline{V} }^{2}}+\frac{1}{2}{\left[1-\frac{16{\overline{D} }^{4}{\overline{v} }_{0}^{4}}{27{\overline{\mu }}^{4}{\overline{V} }^{4}}+\frac{64{\overline{D} }^{3}{\overline{v} }_{0}^{3}}{27{\overline{\mu }}^{3}{\overline{V} }^{3}}-\frac{8{\overline{D} }^{2}{\overline{v} }_{0}^{2}}{3{\overline{\mu }}^{2}{\overline{V} }^{2}}\right]}^{1/2}\right).$$

For ${\overline{v} }_{0}=0$, or $\overline{T }=0$, ${\overline{J} }_{GMG}$ simplifies to (5).

Assuming $\overline{\mu }\gg 1$ (low pressure) eliminates the collisional terms and simplifies (16) and (17) to $\overline{v }(\overline{t})\approx(\overline{J }{\overline{t} }^{2})/2+\overline{E }\overline{t }$ and $\overline{x }(\overline{t})\approx (\overline{J }{\overline{t} }^{3})/6+\overline{E }{\overline{t} }^{2}/2$, respectively. Taking those equations and considering $\overline{V }\gg 1$ recovers the general Child-Langmuir (GCL) function^23^, given by [cf. (9)]

22 $${\overline{J} }_{GCL}=\frac{4\sqrt{2}{\overline{V} }^{3/2}}{9{\overline{D} }^{2}}\left(\sqrt{1+\frac{{\overline{v} }_{0}^{2}}{2\overline{V}} }-\sqrt{\frac{{\overline{v} }_{0}^{2}}{2\overline{V}} }\right){\left(\sqrt{1+\frac{{\overline{v} }_{0}^{2}}{2\overline{V}} }+2\sqrt{\frac{{\overline{v} }_{0}^{2}}{2\overline{V}} }\right)}^{2}.$$

FN defines the limit in which field emission dominates, and is recovered for $\overline{V }\ll 1$ to give

23 $${\overline{J} }_{FN}=\left({\overline{V} }^{2}/{\overline{D} }^{2}\right){e}^{-\overline{D }/\overline{V} }.$$

RLD predicts the limit when thermionic emission dominates, $\overline{T }\gg \overline{E }$, and is given by

24 $${\overline{J} }_{RLD}=\frac{9}{4}{\overline{T} }^{2}{e}^{-1/\overline{T} }.$$

### Theoretical analysis and results

**Figure 1:**
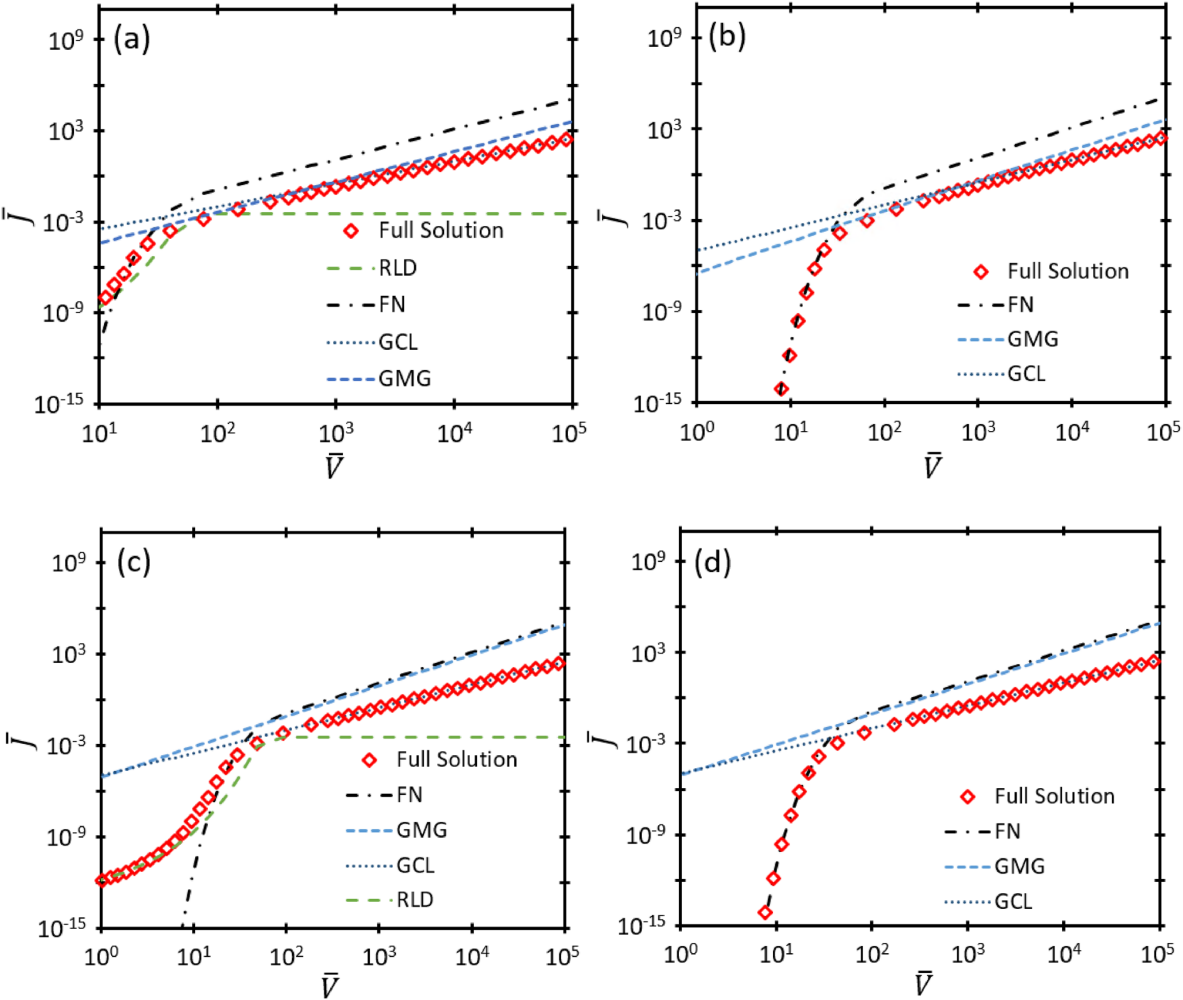
Dimensionless current density $\overline{J }$ as a function of dimensionless voltage $\overline{V }$ for the exact solution represented by (19) and the asymptotic solutions GMG, GCL, FN, and RLD, respectively, with dimensionless gap distance $\overline{D }=250$ for (**a**) dimensionless mobility $\overline{\mu }=5$ and dimensionless temperature $\overline{T }=0.04$; (**b**) $\overline{\mu }=5$ and $\overline{T }=0.002$; (**c**) $\overline{\mu }=100$ and $\overline{T }=0.04$; and (**d**) $\overline{\mu }=100$ and $\overline{T }=0.002$. For a low mobility (or high pressure), the full solution will follow GMG for sufficiently high $\overline{V }$. For a sufficiently high mobility, electron emission bypasses GMG and transitions directly to GCL [cf. (**c**) and (**d**)]. Low temperature eliminates the contribution of thermionic emission, removing the RLD regime from the full solution [cf. (**b**) and (**d**)].

Figure 1 compares the full solution from (19) with the asymptotes defined in (21), (22), (23) and (24) for GMG, GCL, FN, and RLD, respectively, for $\overline{D }=250$. For $\overline{\mu }=5$ and $\overline{T }=0.04$, Fig. 1a shows that electron emission transitions from RLD to FN to GMG to GCL with increasing $\overline{V }$. Reducing $\overline{T }$ to 0.002 for $\overline{\mu }=5$ eliminates the RLD regime in Fig. 1b. In general, for sufficiently low $\overline{\mu }$ in Fig. 1a and b, electron emission first transitions to GMG before reaching GCL. Figure 1c shows that increasing $\overline{\mu }$ to 100 for $\overline{T }=0.04$ causes electron emission to transition from RLD to FN to GCL with increasing $\overline{V }$, bypassing the GMG regime. In addition to demonstrating the direct transition from FN to GCL, Fig. 1d, which considers $\overline{\mu }=100$ and $\overline{T }=0.002$, shows that emission bypasses the RLD regime at low $\overline{V }$, just as for lower $\overline{\mu }$ in Fig. 1b. In all cases, regardless of $\overline{\mu }$, electron emission follows GCL at sufficiently high $\overline{V }$ since the electrons will eventually have enough energy to exhibit vacuum-like behavior in the presence of gas^16,19^. At low $\overline{V }$, electron emission will be driven by RLD at sufficiently high $\overline{T }$ (i.e., ${\overline{v} }_{0}$) or FN at sufficiently low $\overline{T }$ (i.e., ${\overline{v} }_{0}$). Electron emission will only transition to GMG before GCL for a sufficiently low $\overline{\mu }$. The presence or absence of these various transitions may be assessed by using nexus phase space plots.

To characterize the transition between two electron emission mechanisms, we equate $\overline{J }$ from their asymptotic solutions. To simplify GMG and reduce numerical errors, we first derive asymptotic equations in the limits of high (${\overline{v} }_{0}\gg 1$ but still nonrelativistic) and low (${\overline{v} }_{0}\ll 1$, but nonzero) velocity as

25 $${\overline{J} }_{GMG,{\overline{v} }_{0}\gg 1}=\frac{9\overline{\mu }{\overline{V} }^{2}}{8{\overline{D} }^{3}}\left[\frac{16}{9}-\frac{16}{9}\frac{\overline{D }{\overline{v} }_{0}}{\overline{\mu }\overline{V} }-\frac{16}{27}{\left(\frac{\overline{D }{\overline{v} }_{0}}{\overline{\mu }\overline{V} }-1\right)}^{2}\right]$$

and

26 $${\overline{J} }_{GMG,{\overline{v} }_{0}\ll 1}=\frac{9\overline{\mu }{\overline{V} }^{2}}{8{\overline{D} }^{3}}\left(1-\frac{4}{3}\frac{{\overline{D} }^{2}{\overline{v} }_{0}^{2}}{{\overline{\mu }}^{2}{\overline{V} }^{2}}\right),$$

respectively. Setting ${\overline{J} }_{FN}={\overline{J} }_{GMG,{\overline{v} }_{0}\ll 1}$ yields

27 $$\overline{V }=\frac{\overline{D}}{\mathrm{ln }\left(\frac{8\overline{D}}{9\overline{\mu } }\right)-\mathrm{ln}\left(1-\frac{4{\overline{D} }^{2}{\overline{v} }_{0}^{2}}{3{\overline{\mu }}^{2}{\overline{V} }^{2}}\right)},$$

$${\overline{J} }_{FN}={\overline{J} }_{RLD}$$ gives

28 $$2\mathrm{ln}\left(\overline{V }\right)-2\mathrm{ln}\left(\overline{D }\right)-\overline{D }/\overline{V }=2\mathrm{ln}(3\overline{T }/2)-1/\overline{T },$$

$${\overline{J} }_{GCL}={\overline{J} }_{FN}$$ results in

29 $$9{\overline{V} }^{2}{e}^{-\overline{D }/\overline{V} }=2\left(\sqrt{2\overline{V }+{\overline{v} }_{0}^{2}}-{\overline{v} }_{0}\right){\left(\sqrt{2\overline{V }+{\overline{v} }_{0}^{2}}+2{\overline{v} }_{0}\right)}^{2},$$

$${\overline{J} }_{GCL}={\overline{J} }_{GMG,{\overline{v} }_{0}\ll 1}$$ gives

30 $$\frac{81\overline{\mu }{\overline{V} }^{2}}{16\overline{D} }\left(1-\frac{4{\overline{D} }^{2}{\overline{v} }_{0}^{2}}{3{\overline{\mu }}^{2}{\overline{V} }^{2}}\right)=\left(\sqrt{2\overline{V }+{\overline{v} }_{0}^{2}}-{\overline{v} }_{0}\right){\left(\sqrt{2\overline{V }+{\overline{v} }_{0}^{2}}+2{\overline{v} }_{0}\right)}^{2},$$

$${\overline{J} }_{RLD}={\overline{J} }_{GCL}$$ yields

31 $$\overline{D }\overline{T}\mathrm{exp }\left(-\frac{1}{2\overline{T} }\right)=\frac{2\sqrt{2}}{9}{\left(\sqrt{2\overline{V }+{\overline{v} }_{0}^{2}}-{\overline{v} }_{0}\right)}^{1/2}\left(\sqrt{2\overline{V }+{\overline{v} }_{0}^{2}}+2{\overline{v} }_{0}\right),$$

and $${\overline{J} }_{GMG,{\overline{v} }_{0}\ll 1}={\overline{J} }_{RLD}$$ gives

32 $$\overline{V}\overline{\mu }=\sqrt{2{\overline{D} }^{3}\overline{\mu }{\overline{T} }^{2}{e}^{-1/\overline{T} }+4{\overline{D} }^{2}{\overline{v} }_{0}^{2}/3},$$

where $\overline{T }\cong 26.1145{\overline{v} }_{0}^{2}$. Since (27), (28), (29), (30), (31), (32) arise from matching asymptotic solutions, they will not perfectly match the exact solution from (19). This is a standard characteristic of matched asymptotic analyses since the assumptions used to obtain each asymptote will inherently conflict with those to determine the other asymptote (or asymptotes for a higher order nexus) at their intersection^5^. However, these nexuses specify a regime where the dominant electron emission mechanisms would be sensitive to any $\overline{D }$, $\overline{V }$, ${\overline{v} }_{0}$, and $\overline{\mu }$.

**Figure 2:**
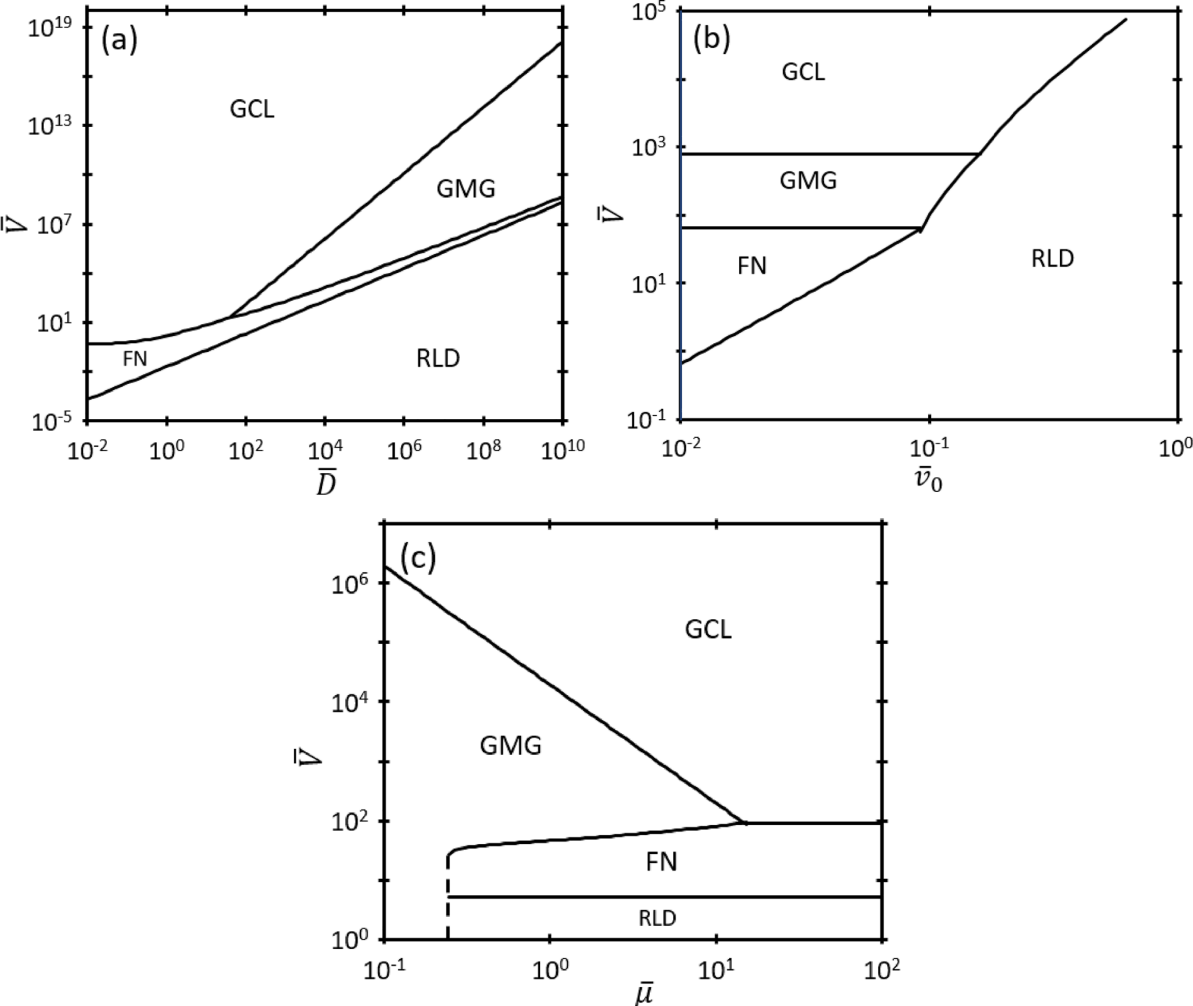
(**a**) Dimensionless breakdown voltage $\overline{V }$ as a function of dimensionless gap distance $\overline{D }$ for dimensionless mobility $\overline{\mu }=5$ and dimensionless temperature $\overline{T }=0.02$. (**b**) Dimensionless breakdown voltage $\overline{V }$ as a function of dimensionless injection velocity ${\overline{v} }_{0}$, for dimensionless mobility $\overline{\mu }=5$ and dimensionless gap distance $\overline{D }=250$. (**c**) Dimensionless breakdown voltage $\overline{V }$ as a function of dimensionless mobility $\overline{\mu }$ for dimensionless temperature $\overline{T }=0.02$ and dimensionless gap distance $\overline{D }=250$.

**Figure 3:**
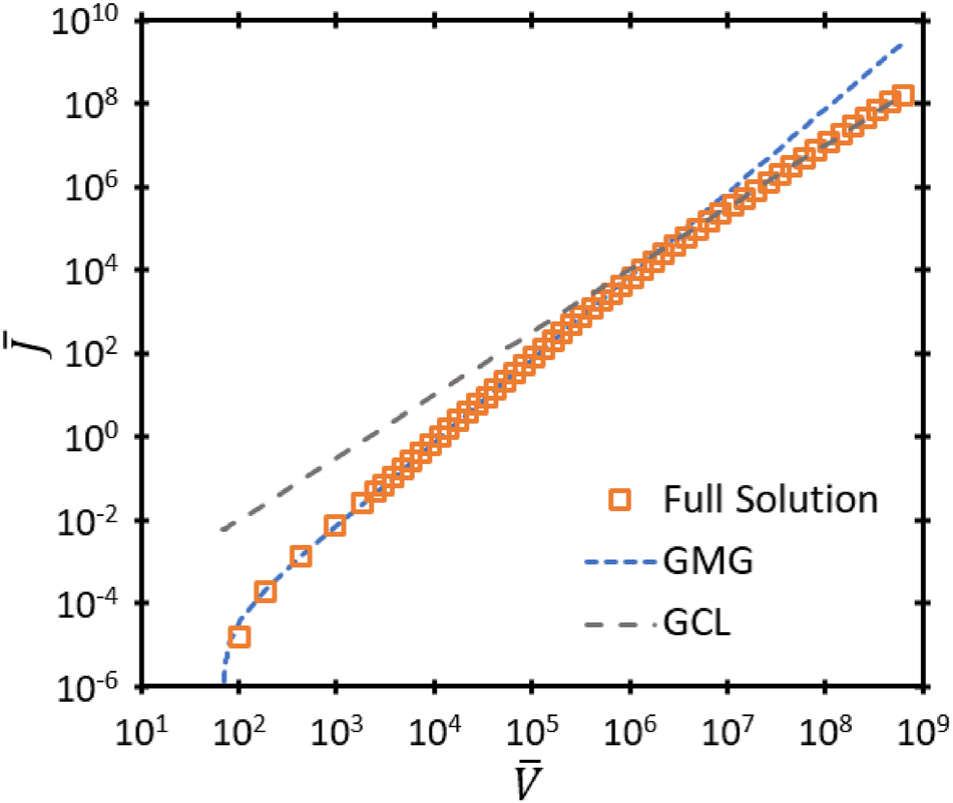
Dimensionless current density $\overline{J }$ as a function of dimensionless voltage $\overline{V }$ for the exact solution represented by (19) and the asymptotic solutions GMG and GCL, respectively, for dimensionless mobility $\overline{\mu }=0.1$, dimensionless gap distance $\overline{D }=250$, and dimensionless temperature $\overline{T }=0.02$. Asymptotic solutions FN and RLD are excluded because they are outside the relevant range.

Figure 2 illustrates the transitions between electron emission mechanisms by plotting $\overline{V }$ as a function of $\overline{D }$, $\overline{\mu }$, and ${\overline{v} }_{0}$. The second-order nexus curves in Fig. 2 demonstrate the conditions where the dominant electron mechanisms transition. RLD consistently dominates when $\overline{V }$ is small, except for $\overline{\mu }\lesssim 0.3$ for $\overline{D }=250$ and $\overline{T }=0.02$, where MG dominates at low $\overline{V }$ because the gap is *always* space-charge limited and ${\overline{J} }_{MG}\approx 0$ for sufficiently low $\overline{V }$ and ${\overline{v} }_{0}\ne 0$. This suggests that combinations of $\overline{\mu }$ and $\overline{V }$ below this threshold (or, alternatively, any pressure *above* this threshold) prohibit the emission of electrons into the gap due to strong collisionality and weak electric field (and concomitant force), respectively, resulting in a near-zero SCLC. Mathematically, for nonzero ${\overline{v} }_{0},$ sufficiently low $\overline{\mu }$ and $\overline{V }$ increases the magnitude of ${\overline{D} }^{4}{\overline{v} }_{0}^{4}{\overline{\mu }}^{-4}{\overline{V} }^{-4}$ such that ${\overline{J} }_{GMG}\to 0$ in (21), making it less than both ${\overline{J} }_{RLD}$ and ${\overline{J} }_{FN}$ so that the gap becomes space-charge limited at any $\overline{V }$ below this threshold $\overline{\mu }$. Increasing $\overline{V }$ eventually causes GCL to dominate at high $\overline{V }$ independent of $\overline{\mu }$ (since ${\overline{J} }_{GMG}$ increases with increasing $\overline{V }$, making it nonzero even at low $\overline{\mu }$), implying that the gap behaves like vacuum for any electron emitted at sufficiently high $\overline{V }$. Figure 3 shows an example of a completely space-charge limited gap where electron emission only transitions from GMG to GCL with no other emission mechanisms involved for $\overline{\mu }=0.1$, $\overline{D }=250$, and $\overline{T }=0.02$.

At least one third-order nexus, corresponding to the intersection of three asymptotic solutions, appears in each panel of Fig. 2. Figures 2a and c show the third-order nexus where ${\overline{J} }_{GMG}={\overline{J} }_{GCL}={\overline{J} }_{FN}$, analogous to our prior study demonstrating the intersection of MG, CL, and FN for zero injection velocity^19^. The third-order nexus between FN, GMG, and GCL informs whether electron emission bypasses the GMG regime during the transition from FN to SCLC with decreasing $\overline{D }$ or increasing $\overline{\mu }$. Figure 2a shows that for $\overline{D }<{\overline{D} }_{GMG,GCL,FN}$ for a fixed ${\overline{v} }_{0}$ and $\overline{\mu }$, electron emission transitions directly from FN to GCL without undergoing GMG, indicating that the gap behaves essentially as vacuum. Figure 2c shows that for $\overline{\mu }>{\overline{\mu }}_{GMG,GCL,FN}$ for a fixed ${\overline{v} }_{0}$ and $\overline{D }$, the electrons encounter sufficiently few collisions crossing the gap such that electron emission transitions directly from FN to GCL without undergoing GMG.

We can derive this condition analytically by rewriting the second-order nexus for ${\overline{J} }_{GCL}={\overline{J} }_{FN}$ as

33 $$\overline{D }=\overline{V}\mathrm{ln }\left[\frac{9{\overline{V} }^{2}}{2{\overline{v} }_{0}^{2}\left(\sqrt{2\overline{V }+{\overline{v} }_{0}^{2}}-{\overline{v} }_{0}\right)+4\overline{V }\left(\sqrt{2\overline{V }+{\overline{v} }_{0}^{2}}+3{\overline{v} }_{0}\right)}\right].$$

The third-order nexus ${\overline{J} }_{GMG}={\overline{J} }_{FN}={\overline{J} }_{GCL}$ can be obtained bv setting ${\overline{J} }_{GMG}={\overline{J} }_{FN}$ and ${\overline{J} }_{GMG}={\overline{J} }_{GCL}$, adding them to obtain $2{\overline{J} }_{GMG}={\overline{J} }_{FN}+{\overline{J} }_{GCL}$, and solving to yield

34 $$\overline{\mu }=\frac{2\overline{D} }{81\overline{V} }\left[9\overline{V}{e }^{-\overline{D }/\overline{V} }+\frac{2{\overline{v} }_{0}^{2}}{\overline{V} }\left(\sqrt{2\overline{V }+{\overline{v} }_{0}^{2}}-{\overline{v} }_{0}\right)+4\sqrt{2\overline{V }+{\overline{v} }_{0}^{2}}+12{\overline{v} }_{0}+\sqrt{2187{\overline{v} }_{0}^{2}+{\left[9\overline{V}{e }^{-\overline{D }/\overline{V} }+\frac{2{\overline{v} }_{0}^{2}}{\overline{V} }\left(\sqrt{2\overline{V }+{\overline{v} }_{0}^{2}}-{\overline{v} }_{0}\right)+4\sqrt{2\overline{V }+{\overline{v} }_{0}^{2}}+12{\overline{v} }_{0}\right]}^{2}} \right].$$

**Figure 4:**
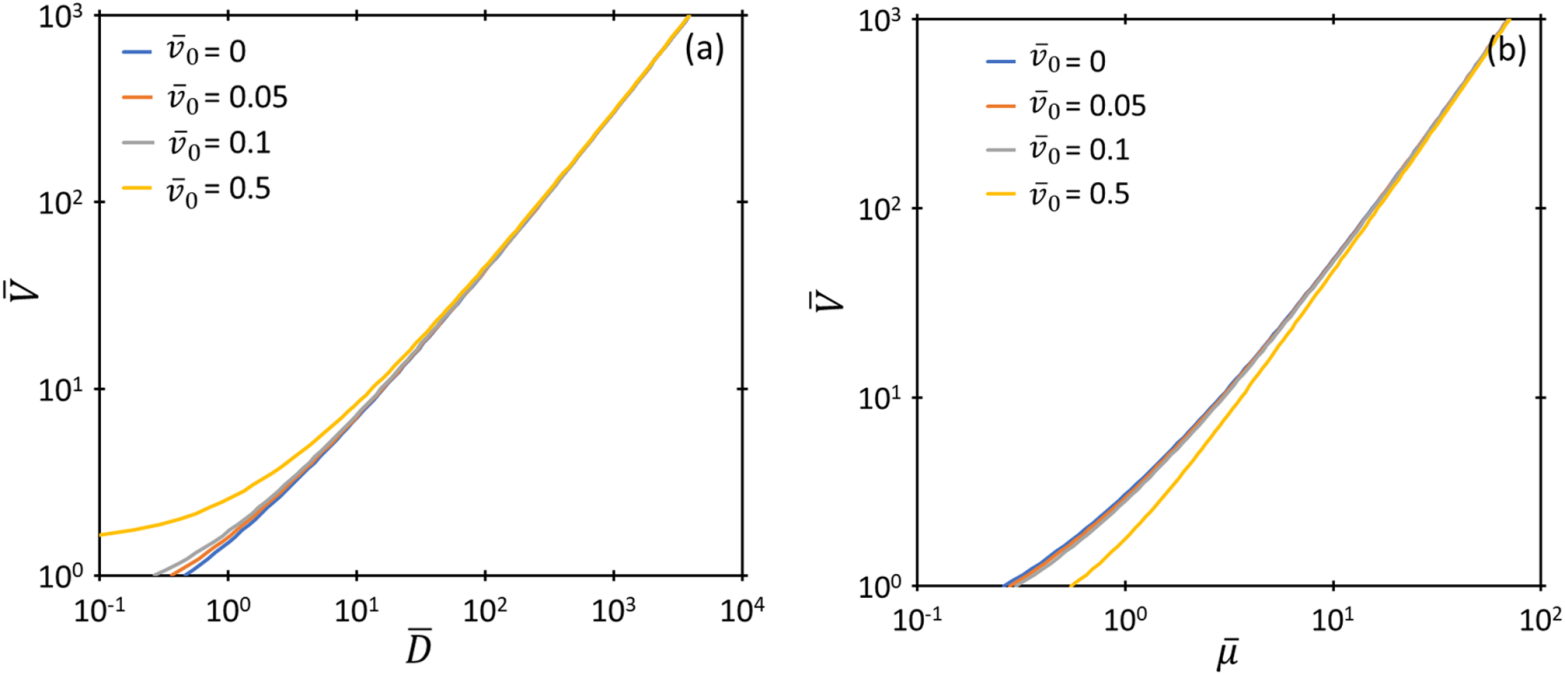
Dimensionless voltage $\overline{V }$ as a function of (**a**) dimensionless gap distance $\overline{D }$ and (**b**) dimensionless mobility $\overline{\mu }$ describing the third-order nexus between GCL, GMG, and FN.

Equations (33) and (34) describe the third-order nexus between FN, GMG, and GCL for some fixed $\overline{D }, \overline{V },$ or $\overline{\mu }$ for a given ${\overline{v} }_{0}$. Comparing the solution from (33) to (34) to the solution for zero injection velocity^19^ shows that incorporating ${\overline{v} }_{0}$ induces a negligible change in the third-order nexus (< 0.1%) for ${10}^{5}\le \overline{V}\le {10 }^{15}$; hence, we may use the zero injection nexus relationships, given by

35 $${\overline{D} }_{GMG,GCL,FN}=\overline{V}\mathrm{ln }\left[\frac{9\sqrt{\overline{V}}}{4\sqrt{2} }\right]$$

and

36 $${\overline{\mu }}_{GMG,GCL,FN}={\overline{D} }_{GMG,GCL,FN}\left(\frac{16\sqrt{2}}{81\sqrt{\overline{V}} }+\frac{4}{9}{e}^{-{\overline{D} }_{GMG,GCL,FN}/\overline{V} }\right)$$

for the given voltage range with minimal loss of accuracy, as done previously when incorporating thermionic emission^23^. Figure 3 from^19^ shows the behavior of the third-order nexus under this condition. The impact of injection velocity on the third-order does not become significant until $\overline{V }<{10}^{3}$, as shown in Fig. 4, which shows how $\overline{V }$ at the third-order nexus behaves as a function of $\overline{D }$ and $\overline{\mu }$ for different ${\overline{v} }_{0}$, respectively. The divergence due to nonzero ${\overline{v} }_{0}$ becomes particularly pronounced at low $\overline{V }$ for both low $\overline{D }$ and $\overline{\mu }$.

Two third-order nexuses arise when considering $\overline{V }$ as a function of ${\overline{v} }_{0}$ in Fig. 2b: one nexus between RLD, FN, and GMG and a second between RLD, GMG, and GCL. This indicates that for a given ${\overline{v} }_{0}$, the full solution transitions from RLD to FN to GCL, which is demonstrated in Fig. 1, or transitions from RLD to GMG to GCL, bypassing the FN regime. For the third-order nexus ${\overline{J} }_{GMG}={\overline{J} }_{FN}={\overline{J} }_{RLD}$, the condition can be derived analytically by considering ${\overline{J} }_{FN}={\overline{J} }_{RLD}$ to obtain

37 $$\overline{D }=2\overline{V }W\left[\frac{1}{3\sqrt{{e}^{-1/\overline{T} }{\overline{T} }^{2}}}\right],$$

where $W$ is the Lambert W-function, or the product log function. The third-order nexus can then be recovered by setting ${\overline{J} }_{GMG}={\overline{J} }_{FN}$ and ${\overline{J} }_{FN}={\overline{J} }_{RLD}$, adding them to obtain $2{\overline{J} }_{FN}={\overline{J} }_{RLD}+{\overline{J} }_{GMG}$, and solving to yield

38 $$\overline{\mu }=\frac{1}{9{\overline{V} }^{2}}\left(8\overline{D }{\overline{V} }^{2}{e}^{-\overline{D }/\overline{V} }-9{\overline{D} }^{3}{\overline{T} }^{2}{e}^{-1/\overline{T} }-\sqrt{108{\overline{D} }^{2}{\overline{v} }_{0}^{2}{\overline{V} }^{2}+{\left(9{\overline{D} }^{3}{\overline{T} }^{2}{e}^{-1/\overline{T} }-8\overline{D }{\overline{V} }^{2}{e}^{-\overline{D }/\overline{V} }\right)}^{2}}\right).$$

Similarly, the condition for the third-order nexus ${\overline{J} }_{GCL}={\overline{J} }_{GMG}={\overline{J} }_{RLD}$ can be derived analytically by first considering ${\overline{J} }_{GCL}={\overline{J} }_{RLD}$ to obtain

39 $$\overline{D }=\frac{2\sqrt{2}}{9\overline{T}}\mathrm{exp }\left(\frac{1}{2\overline{T} }\right)\sqrt{{\left({\overline{v} }_{0}^{2}+2\overline{V }\right)}^{3/2}+6\overline{V }{\overline{v} }_{0}-{\overline{v} }_{0}^{3}},$$

where $\overline{T }\propto {\overline{v} }_{0}^{2}$. As above, considering ${\overline{J} }_{GMG}={\overline{J} }_{RLD}$ and ${\overline{J} }_{GCL}={\overline{J} }_{GMG}$, adding them to obtain $2{\overline{J} }_{GMG}={\overline{J} }_{GCL}+{\overline{J} }_{RLD}$, and solving for $\overline{\mu }$ gives

40 $$\overline{\mu }=\frac{\overline{D} }{162{\overline{V} }^{2}}\left(81{\overline{D} }^{2}{\overline{T} }^{2}{e}^{-1/\overline{T} }+8{\left(2\overline{V }+{\overline{v} }_{0}^{2}\right)}^{3/2}+48\overline{V }{\overline{v} }_{0}-8{\overline{v} }_{0}^{3}+\sqrt{34992{\overline{v} }_{0}^{2}{\overline{V} }^{2}+{\left(81{\overline{D} }^{2}{\overline{T} }^{2}{e}^{-1/\overline{T} }+8{\left(2\overline{V }+{\overline{v} }_{0}^{2}\right)}^{3/2}+48\overline{V }{\overline{v} }_{0}-8{\overline{v} }_{0}^{3}\right)}^{2}}\right).$$

Although not visible in Fig. 2, a fourth-order nexus may also occur between RLD, FN, GMG, and GCL for an appropriate combination of $\overline{V }, \overline{D }, \overline{\mu },$ and ${\overline{v} }_{0}$. Selecting any one of these parameters uniquely defines the other three to achieve the fourth-order nexus. To predict the fourth-order nexus, we first determine $\overline{D }$ with respect to ${\overline{v} }_{0}$ and $\overline{V }$. Since ${\overline{J} }_{RLD}={\overline{J} }_{FN}={\overline{J} }_{GMG}={\overline{J} }_{GCL}$, we can solve for $\overline{D }$ independent of $\overline{\mu }$ by considering ${\overline{J} }_{RLD}={\overline{J} }_{GCL}$, yielding $\overline{D }$ by (39). The fourth-order nexus can then be recovered by setting ${\overline{J} }_{GMG}={\overline{J} }_{GCL}, {\overline{J} }_{GMG}={\overline{J} }_{FN}$ and ${\overline{J} }_{GMG}={\overline{J} }_{RLD}$, adding them to obtain $3{\overline{J} }_{GMG}={\overline{J} }_{GCL}+{\overline{J} }_{RLD}+{\overline{J} }_{FN}$, and solving for $\overline{\mu }$ to obtain

41 $$\overline{\mu }=\frac{\overline{D} }{243{\overline{V} }^{2}}\left[81{\overline{D} }^{2}{\overline{T} }^{2}{e}^{-1/\overline{T} }+36{\overline{V} }^{2}{e}^{-\overline{D }/\overline{V} }+8{\left({\overline{v} }_{0}^{2}+2\overline{V }\right)}^{3/2}+48{\overline{v} }_{0}\overline{V }-8{\overline{v} }_{0}^{3}+\sqrt{78732{\overline{v} }_{0}^{2}{\overline{V} }^{2}+{\left[81{\overline{D} }^{2}{\overline{T} }^{2}{e}^{-1/\overline{T} }+36{\overline{V} }^{2}{e}^{-\overline{D }/\overline{V} }+8{\left({\overline{v} }_{0}^{2}+2\overline{V }\right)}^{3/2}+48{\overline{v} }_{0}\overline{V }-8{\overline{v} }_{0}^{3}\right]}^{2}}\right].$$

**Figure 5:**
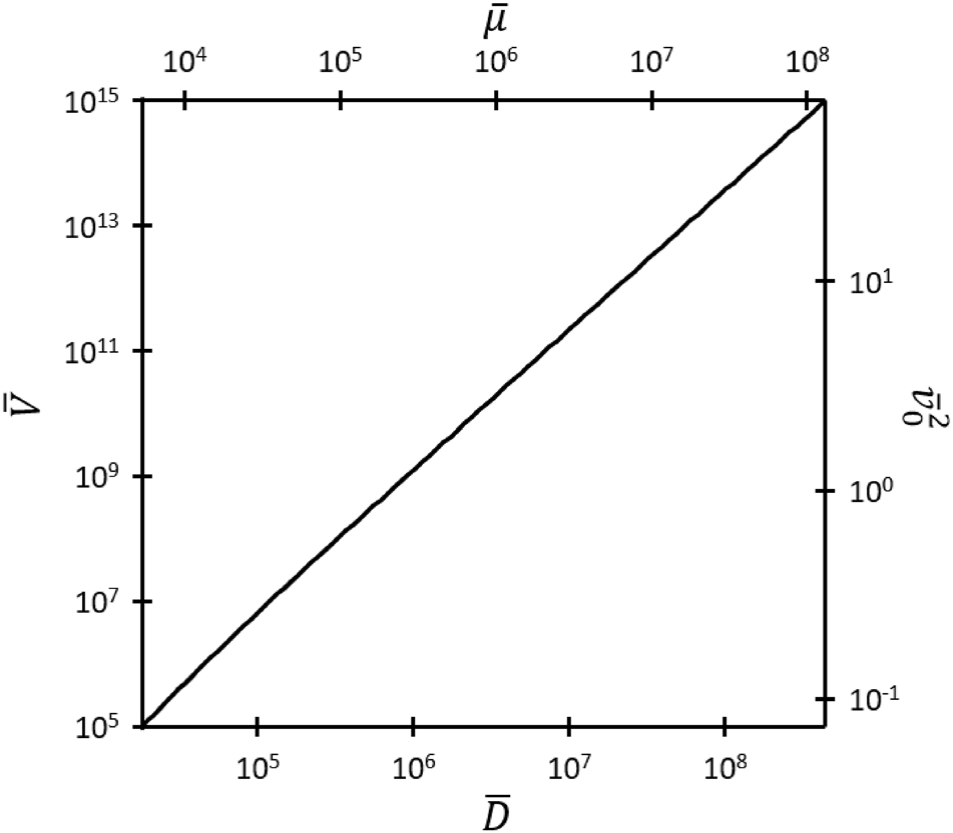
The fourth-order nexus corresponding to ${\overline{J} }_{RLD}={\overline{J} }_{FN}={\overline{J} }_{GMG}={\overline{J} }_{GCL}$ uniquely defined by any one of the dimensionless voltage $\overline{V }$, dimensionless injection velocity squared ${\overline{v} }_{0}^{2}$, dimensionless gap distance $\overline{D }$, or dimensionless mobility $\overline{\mu }$.

Equations (39) and (41) describe the fourth-order nexus between RLD, FN, GMG, and GCL for a combination of $\overline{D }, \overline{V },$ $\overline{\mu }$, or ${\overline{v} }_{0}$, shown in Fig. 5.

**Figure 6:**
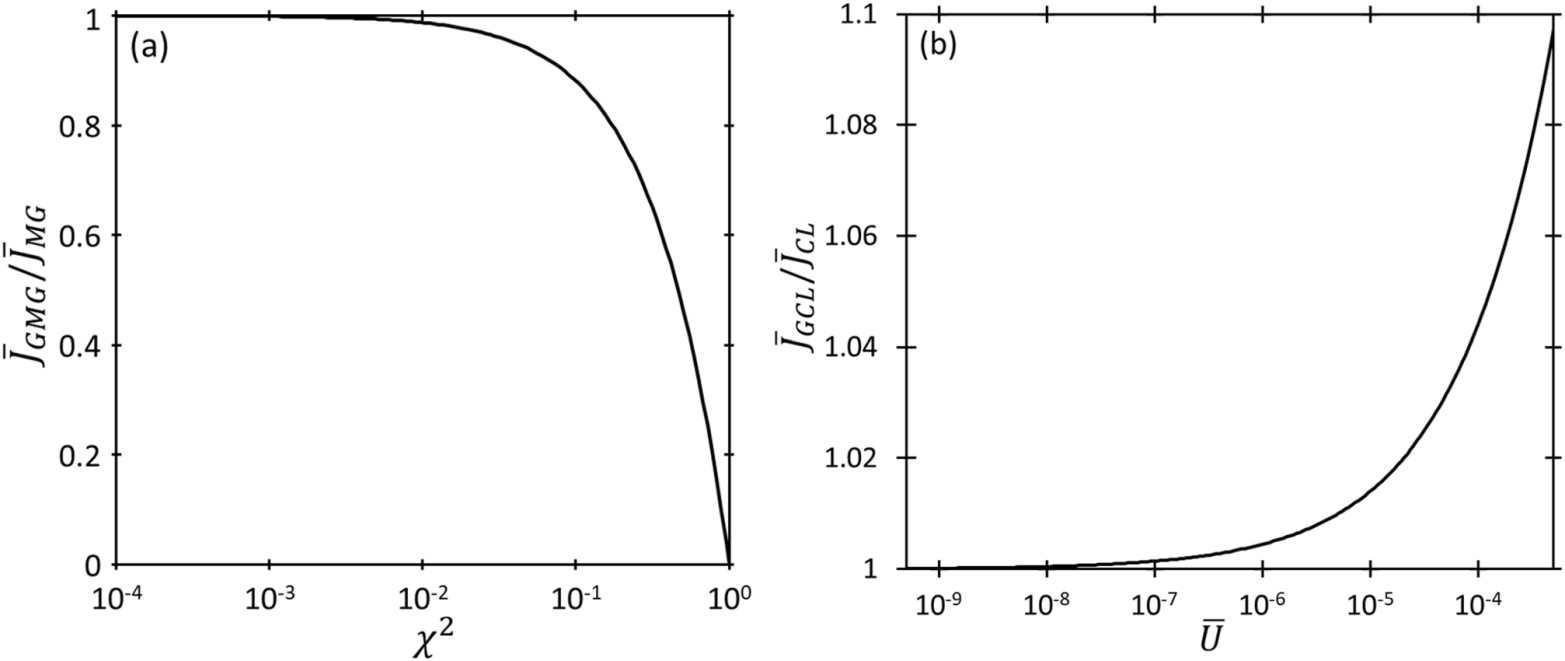
(**a**) Ratio of dimensionless GMG current density ${\overline{J} }_{GMG}$ to MG current density ${\overline{J} }_{MG}$ as a function of ${\chi }^{2}=[{\overline{D }{\overline{v} }_{0}/\left(\overline{\mu }\overline{V }\right)]}^{2}$. (**b**) Ratio of dimensionless GCL current density ${\overline{J} }_{GCL}$ to CL current density ${\overline{J} }_{CL}$ as a function of the ratio of kinetic energy (KE) to potential energy (PE), given by $\overline{U }$.

Figure 6 compares the two space-charge-limited regimes, GCL and GMG, by plotting each with respect to the corresponding equation with zero injection velocity. From above, GMG differs from MG by a recurring term ${\chi }^{2}=[{\overline{D }{\overline{v} }_{0}/\left(\overline{\mu }\overline{V }\right)]}^{2}$, which represents the ratio of initial velocity ${\overline{v} }_{0}$ to the nominal drift velocity ${\overline{v} }_{D}=\overline{\mu }\overline{V }/\overline{D }$; therefore, we plot ${\overline{J} }_{GMG}/{\overline{J} }_{MG}$ as a function of ${\chi }^{2}$ in Fig. 6a. This demonstrates that ${\overline{J} }_{GMG}$ decreases and approaches zero with increasing ${\chi }^{2}$, which corresponds to ${\overline{v} }_{d}\ll {\overline{v} }_{0}$. This means that insufficient electric field and/or low mobility ($\overline{\mu }\to 0$, which corresponds to a strongly collisional gap) makes it difficult for the electrons to move in the medium. Since ${\overline{J} }_{GMG}\to 0$, as ${\chi }^{2}\to \infty$, the gap will always be space-charge limited since its strong collisionality or weak electric field prohibits electron emission until the applied voltage becomes sufficiently high, at which point the gap becomes space-charge limited by CL [cf. Fig. 2c]. With decreasing ${\chi }^{2}$ (i.e., ${\overline{v} }_{d}\ll {\overline{v} }_{0}$), ${\overline{J} }_{GMG}\to {\overline{J} }_{MG}$. Figure 6b shows ${\overline{J} }_{GCL}/{\overline{J} }_{CL}$ as a function of $\overline{U }={\overline{v} }_{0}^{2}/\left(2\overline{V }\right)$, which is the standard scaling for CL with nonzero ${\overline{v} }_{0}$ for planar^49–51^ and nonplanar^53,54^ diodes. As $\overline{U }\to 0$, ${\overline{J} }_{GCL}\to {\overline{J} }_{CL}$; as $\overline{U }\to \infty$, ${\overline{J} }_{GCL}$ continues to increase. At some point, relativistic effects become important^55^, which is beyond the scope of the current study.

## Conclusion

This paper extends nexus theory to assess the transitions between RLD, FN, GMG, and GCL. Nexus theory provides a way to assess the dominant mechanisms before carrying out complicated simulations or experiments. Constructing the nexus phase space plot using the analytic equations for these mechanisms (including the GMG derived in this manuscript) with the desired operating conditions will demonstrate whether the equation for a single mechanism can be used (i.e., well away from one of the nexus curves) or if a more complicated equation combining multiple equations must be used (i.e., close to a nexus curve for two or more mechanisms). If this simple analysis shows that only one of these equations is dominant, this simplifies the design and simulation of the specific device; however, if operating near a nexus, then one must combine the mechanisms and use a more complete theory to predict behavior. The difference between using one of the simple equations can be significant, as illustrated by the 153.4% difference between GMG and the exact solution and 96.0% difference between FN and the exact solution in plot a of Fig. 1 at $\overline{V }=25.73687$ near the nexus curve between FN and GMG.

The exact solution that accounts for temperature and mobility approaches the accepted equations for these mechanisms in the appropriate limits. Furthermore, we derive an analytic equation for GMG that includes the injection velocity, which is relevant for thermionic emitters under non-vacuum conditions. We also observe that applying a sufficiently small (but nonzero) bias voltage with a sufficient mobility yields a near-zero ${\overline{J} }_{GMG}$ such that the gap is *always* space-charge limited, ultimately transitioning to GCL with increasing bias voltage.

We have recently experimentally and theoretically assessed the transitions between FN, MG, CL, and field emission-driven breakdown for nanoscale gaps at atmospheric pressure^7^ and vacuum^56^ near the third-order nexus between FN, MG, and CL. Other theories have examined thermo-field emission driven breakdown for microscale gaps at microwave frequencies^57,58^, indicating that the electrons undergoing these mechanisms need not only originate from cold cathodes. Thus, the results reported here, particularly for the fourth-order nexus between RLD, FN, GMG, and GCL, elucidate the contributions of the emission mechanisms (RLD and FN) and limits (GMG and GCL) for thermionic emitters in nano- and microscale devices that may undergo gas breakdown. Such theories may ultimately be extended to include additional emission mechanisms, such as photoemission^34^, or nonplanar geometries, which have been an ongoing area of study for both zero^59^ and nonzero^53,54^ injection velocity. Meadors and Poirier studied how to use a laser to heat a cathode to induce thermionic emission in vacuum and at atmospheric pressure without electromagnetic interference^60^. Another study addressed cathode heating and subsequent thermionic emission that play a critical role in arc formation^61^. Go^62^ pointed out that ion-enhanced thermo-field emission enabled the study of how slow-moving ions influenced thermionic emission in cathodic arcs^63–67^ and then developed a theory for thermo-field emission driven microscale breakdown, extending the typi cal theories that considered strictly field emission driven breakdown^5^. As gap distances become smaller, the operating conditions approach the regime where the individual RLD, FN, MG, and CL may not capture the physics and a combined theory as derived in this paper becomes necessary.

Mobility may also be incorporated into recent work that unified RLD, FN, and SCLC in a vacuum crossed-field diode, where a magnetic field is applied perpendicular to the applied electric field^68^. All these applications of nexus theory demonstrate the importance of appropriately characterizing the dominant mechanism(s) to determine the current density. While perhaps not as important when considering two conditions (e.g., FN and CL), this becomes increasingly important as more mechanisms are added and the phase plot of contributing variables (e.g., voltage, gap distance, pressure, and temperature) increases. This complicates the transitions between the mechanisms and necessitates more care for ensuring that any theories or simulations properly account for the dominant mechanism.

Finally, we point out that the present paper considers the emission mechanism(s) predicted by the theory as coming from the full device. In other words, we do not consider that nonuniformities in temperature or electric field may result in different areas of the emitter undergoing emission mechanisms. Such a multidimensional model may be interesting to better understand behavior in these different regimes in future studies, but the approach here provides value for experimentalists to rapidly characterize the overall behavior of the measured current–voltage plots of the overall device.

### Supplementary Information

Supplementary Information.

## Data Availability

All data generated or analyzed during this study are included in this published article. This paper reports the results from theoretical research.
