# Supplementary material for: The transition from field emission to collisional space-charge limited current with nonzero initial velocity
Source: Sci Rep. 2023 Sep 4;13:14505. doi: 10.1038/s41598-023-41615-2 (PMC10477287; doi:10.1038/s41598-023-41615-2)
Supplement: Supplementary file 1 — Supplementary Information. [file 41598_2023_41615_MOESM1_ESM.docx]

***Supplementary Information:* Incorporating Electron Injection Velocity into the Transition from Field Emission to Space-Charge Limited Emission with Collisions**

Lorin I. Breen,^1,2^ Amanda M. Loveless,^3^ Adam M. Darr,^3^ Keith L. Cartwright,^4^ and Allen L. Garner^2,3,5Ass^

^1^*School of Health Sciences, Purdue University, West Lafayette, Indiana 47907, USA*

^2^*Department of Agricultural and Biological Engineering, Purdue University, West Lafayette, Indiana 47907, USA*

^3^*School of Nuclear Engineering, Purdue University, West Lafayette, Indiana 47907, USA*

*^4^Sandia National Laboratories, Albuquerque, New Mexico 87123, USA*

*^5^Elmore Family School of Electrical and Computer Engineering, West Lafayette, Indiana 47907 USA*

Correspondence and requests for materials should be addressed to A. L. G (email: [algarner@purdue.edu](mailto:algarner@purdue.edu))

In the main manuscript, we use the generalized thermal-field (GTF) emission theory as the canonical current density source combining thermionic and field emission. This Supplementary Information details the equations necessary to fully define the GTF theory.

These following equations extend the general-thermal-field model and are used throughout the paper [23]:

| $y=\frac{\sqrt{4QF}}{\Phi},$ | (S1) |
| --- | --- |
| $v\left( y \right)=1-\frac{y^{2}}{3}\left[ 3-\ln\left( y \right) \right], t\left( y \right)=1+\frac{y^{2}}{9}\left[ 1-\ln\left( y \right) \right],$ | (S2) |
| $\phi=\left( 1-y \right)\Phi,$ | (S3) |
| $T_{min}=\frac{\hbar F}{2k_{B}t(y)\sqrt{2m\Phi}},$ | (S4) |
| $T_{max}=\frac{\hbar F}{\pi k_{B}\sqrt{m\Phi y}}.$ | (S5) |

When $T<T_{min},$

| $\beta_{F}=\frac{2t(y)}{\hbar F}\sqrt{2m\Phi}, s=\frac{4v(y)\sqrt{2m\Phi^{3}}}{3\hbar F}.$ | (S6) |
| --- | --- |

When $T>T_{max}$,

| $\beta_{F}=\frac{\pi\sqrt{m\Phi y}}{\hbar F}, s=\frac{\pi\sqrt{m\Phi y}}{\hbar F}\phi.$ | (S7) |
| --- | --- |

When $T>T_{min}$ and $T<T_{max}$, $n=1$, making $\beta_{F}=\beta_{T}$ and

| $s=\beta_{T}\left( E_{m}+\frac{\theta\left( E_{m} \right)}{\beta_{F}\left( E_{m} \right)}-\mu^{*} \right),$ | (S8) |
| --- | --- |

where $\mu^{*}$ is the Fermi energy and $E_{m}$ can be found from

| $\beta_{T}=\beta_{F}(E_{m}),$ | (S9) |
| --- | --- |

where

| $\beta_{F}\left( E \right)\approx\frac{1}{\phi}\left[ B_{q}z+C_{FN}\left( 1-z \right)+3\left( 2B_{FN}-B_{q}-C_{FN} \right)z\left( 1-z \right) \right],$ | (S10) |
| --- | --- |

and

| $\theta\left( \mu+z\phi\right)=B_{FN}-C_{FN}z+z^{2}\left[ \left( C_{FN}-C_{q} \right)\left( 2-z \right)-\left( B_{FN}-B_{q} \right)\left( 3-2z \right) \right],$ | (S11) |
| --- | --- |

with

| $B_{FN}=\frac{4v\left( y \right)\sqrt{2m\Phi^{3}}}{3\hbar F}, C_{FN}=\frac{2t(y)}{\hbar F}\sqrt{2m\Phi}, B_{q}=C_{q}=\frac{\pi\sqrt{2m}}{\hbar}\phi\left( \frac{Q}{F^{3}} \right)^{\frac{1}{4}}.$ | (S12) |
| --- | --- |

Once the necessary values are found, they can be plugged into

| $N\left( n,s \right)=n\int_{-\infty}^{\infty} \frac{\ln\left[ 1+e^{n\left( k-s \right)} \right]}{1+e^{k}}dk.$ | (S13) |
| --- | --- |

[23] Darr, A.M., Darr, C. R. & Garner, A. L. Theoretical assessment of transitions across thermionic, field, and space-charge limited emission. *Phys. Rev. Res*. **2**, 033137 (2020).
